# A Comparison of Machine Learning Models for Classification of Parkinson’s Disease During a Working Memory and Sustained Attention Task

**DOI:** 10.3390/brainsci16080781

**Published:** 2026-07-24

**Authors:** Mercedes A. Terry, Samuel A. Birkholz, Jeffrey S. Johnson, Jau-Shin Lou, Asenath X. A. Huether, Jessica Keller, Enrique Alvarez-Vazquez

**Affiliations:** 1 Biomedical Engineering Department, University of North Dakota, Grand Forks, ND 58202, USA; enrique.vazquez@und.edu; 2 Department of Psychology, North Dakota State University, Fargo, ND 58102, USA; samuel.birkholz@sanfordhealth.org (S.A.B.); jeffrey.s.johnson@ndsu.edu (J.S.J.); 3 Department of Neurology, Sanford Health, Fargo, ND 58103, USA; jau-shin.lou@sanfordhealth.org (J.-S.L.); jessica.keller@sanfordhealth.org (J.K.); 4 Department of Neurology, University of North Dakota School of Medicine and Health Science, Grand Forks, ND 58202, USA; 5 Department of Psychology, Minnesota State Community and Technical College, Moorhead, MN 56560, USA; asenath.huether@minnesota.edu

**Keywords:** cognitive biomarkers, electroencephalography (EEG), machine learning, multimodal classification, Parkinson’s disease, pupillometry, radial basis function support vector machine (SVM-RBF), sustained attention, task-based biomarkers, working memory

## Abstract

**Highlights:**

**What are the main findings?**

- A task-aligned machine learning pipeline combining EEG and pupillometry features separated individuals with Parkinson’s disease from healthy controls during a working memory task, achieving accuracy that exceeded what standard behavioral performance measures alone could distinguish between the two groups.
- Seven physiological features, including markers of frontal alpha activity, oscillatory ratios, and pupillary response, emerged as most discriminative, concentrated in the most demanding parts of the task (higher memory loads, longer delays, and incorrect trials).

**What are the implications of the main findings?**

- Physiological signals recorded during cognitive tasks may capture Parkinson’s-related cognitive changes that are not reliably evident from behavioral performance alone, pointing to their potential value as more sensitive early markers of cognitive dysfunction.
- The task-aligned, interpretable design of this framework offers a reproducible platform for future biomarker discovery, laying groundwork for larger validation studies and, eventually, tools to support cognitive screening and monitoring in Parkinson’s disease.

**Abstract:**

Background and Objectives: Individuals with Parkinson’s disease (PD) experience deficits in working memory (WM) and sustained attention (ATTN), but diagnosing and monitoring these deficits remains challenging. This study compares machine learning (ML) classification models trained on EEG and pupillometry data from WM and ATTN tasks to identify task-specific and shared cognitive biomarkers of PD. Methods: EEG and pupillometry were recorded from PD patients and healthy controls (HC) during a visual change detection WM task and a continuous performance ATTN task. A standardized toolbox extracted 108 features, reduced via PCA and recursive elimination (RE) and classified using an SVM-RBF within a nested, 5-fold cross-validated pipeline, with class balancing (SMOTE) and feature selection performed strictly within training folds to prevent leakage. Results: On internal test folds, WM achieved 71% accuracy (F1 = 0.701) and ATTN achieved 73% (F1 = 0.699); on an independent hold-out set, WM achieved 63% accuracy (F1 = 0.626) and ATTN achieved 70% (F1 = 0.623). ATTN showed higher accuracy and precision, WM showed higher recall, and univariate analyses independently supported several top-ranked features (e.g., theta/beta and alpha/theta ratios); PAI and FAA, though top features in both tasks, reached univariate significance only in ATTN. These results indicate WM and ATTN yield complementary, task-linked neurophysiological signatures relevant to PD classification. Conclusion: At its current stage, this pipeline functions as a research tool for biomarker discovery rather than a clinical diagnostic, though larger, externally validated samples could support future screening and monitoring applications.

## 1. Introduction

Parkinson’s disease (PD) is a progressive neurodegenerative disease that affects more than 10 million people worldwide and is characterized primarily by outward motor symptoms, such as bradykinesia, tremor, and rigidity, resulting from the degeneration of dopaminergic neurons in the substantia nigra and related basal ganglia circuits [1]. Although PD is traditionally defined and evaluated by its motor presentation, non-motor symptoms such as deficits in cognition are increasingly recognized as critical determinants of patient quality of life and functional independence [2,3,4].

Cognitive dysfunction in PD commonly involves slowed processing speed, impaired executive function, reduced working memory (WM) capacity, and deficits in sustained attention (ATTN) [3,4,5,6,7]. Such impairments may appear early in the disease and progress to mild cognitive impairment or dementia [7], where up to 80% of individuals with longer disease duration develop Parkinson’s disease dementia [8]. Cognitive decline also contributes to increased caregiver burden, as patients may require assistance with activities of daily living and decision-making [9,10]. Furthermore, cognitive impairments in PD are associated with reduced quality of life [6,11,12]. Importantly, the cognitive profile in PD is heterogeneous, with some patients demonstrating predominant deficits in attention and executive function while others exhibit more pronounced memory impairment [5,8,13]. This variability complicates early detection and underscores the need for sensitive, task-based measures that can identify subtle changes before overt clinical decline [14]. The classification of cognitive subtypes, including amnestic, non-amnestic, single-domain, and multi-domain mild cognitive impairment profiles, highlights distinct cognitive patterns that warrant the use of tailored assessment tools [13]. Furthermore, more recent data-driven studies have identified memory-predominant versus visuospatial-predominant dementia subtypes in PD, each associated with distinct neurobiological and clinical characteristics [15]. Considering this heterogeneity, research has begun to explore novel cognitive-control-based tasks that are more sensitive than standard cognitive assessments for detecting early or subtle deficits [14]. The complexity and variability of cognitive dysfunction in PD make it challenging to assess using conventional methods, highlighting the potential of machine learning (ML) to enhance early detection and prediction of cognitive decline [16].

ML approaches have been increasingly applied to cognitive dysfunction detection in PD using EEG and pupillometry [16]. Support vector machines (SVMs) have been widely used and have been shown to achieve high classification accuracy when trained on spectral, power, connectivity, and ERP features [17,18,19,20,21]. For example, Anjum et al. [17] reported that resting-state EEG spectral and entropy features classified PD with high accuracy using SVM and random forest models. Srinivasan et al. [22] demonstrated that combining theta and alpha band features from an n-back task with an SVM yielded significant classification accuracy, highlighting the diagnostic value of cognitive task-based EEG classification. Deep learning frameworks, such as convolutional neural networks (CNNs), have also been used to extract spatial–temporal EEG features and achieve comparable or superior performance [22]. Beyond resting-state data, task-evoked EEG studies have leveraged ERPs and time-frequency measures from WM and ATTN paradigms to improve sensitivity to cognitive impairment in PD [23].

Pupillometry-based ML studies have also been used in PD classification models; however, fewer works focused on pupillometry features than on EEG [16,24]. Existing work has primarily examined autonomic and cognitive workloads, such as mean pupil diameter, dilation velocity, and blink metrics, for classifying PD [24,25]. For example, Kahya et al. [24] found that pupil dilation dynamics during attention-demanding tasks correlated with PD severity, and feature-based logistic regression models achieve significant accuracy. Qian et al. [26] reported that the multimodal fusion of pupillometry and EEG improves classification over using either modality alone.

Compared to deep learning methods such as CNNs or decision tree-based methods (DTs), SVMs often offer superior performance in small to medium-sized datasets, which are typical in PD cognitive research, due to their ability to find optimal separating hyperplanes in high-dimensional feature spaces without overfitting [27,28,29]. SVMs are also less data-hungry than CNNs, making them more suitable for EEG and pupillometry studies with limited participant numbers [30]. In contrast to DTs, which can be unstable with small perturbations in training data, SVMs maintain robust generalization by focusing on the most informative samples [28,31]. This makes SVMs particularly well-suited for multimodal, high-dimensional feature sets such as those extracted from EEG and pupillometry, where interpretability and stability are critical for clinical translation. The use of a radial basis function (RBF) kernel with SVM further enhances performance by enabling the model to capture nonlinear relationships between features and class labels, making it effective for complex neurophysiological data [32,33].

However, most of these models have focused on single tasks or modalities, limiting insights into how task demands influence discriminative feature patterns [34]. Many also rely on relatively small feature sets, often limited to a handful of spectral or ERP metrics, which can constrain the richness of the information available for classification and reduce generalizability across datasets [16,35]. Additionally, these models often lack interpretability due to the black box nature of ML models, making it difficult to link predictive features to known neurophysiological processes [34,36]. In addition, models fail to extract features temporally aligned with task events, potentially missing transient cognitive and physiological responses critical for distinguishing PD from healthy controls (HC) [37]. Aligning feature extraction with stimulus presentation, retention intervals, and response periods could provide greater sensitivity to specific cognitive deficits [38]. Lastly, these models fail to directly compare models across multiple cognitive paradigms using the same participants, which limits their generalizability beyond a single task [39]. The key limitations in current ML models, including a small feature set, a lack of temporal alignment with task events, and insufficient cross-task comparisons, constrain their effectiveness for diagnostics and ongoing cognitive monitoring [36].

The primary contributions of this study are fourfold. First, we apply a substantially larger and more diverse feature set (108 EEG and pupillometry features spanning ten domains) than is typical in PD cognitive-classification studies, which commonly rely on a narrow set of spectral or ERP metrics. Second, we directly compare classification performance across two distinct cognitive paradigms (WM and ATTN) using an identical feature-extraction and modeling pipeline in the same participants, which prior work has not done, allowing task-driven differences in model behavior to be isolated from pipeline differences. Third, by temporally aligning feature extraction with task-relevant bins (e.g., encoding vs. retrieval, set size, delay duration), we link classification-relevant signals to specific cognitive processing stages rather than treating each recording as a single undifferentiated epoch. Fourth, we identify two features, frontal alpha asymmetry (FAA) and the prefrontal alpha index (PAI), that emerge as top contributors in both tasks, providing preliminary evidence for candidate cross-task neurophysiological markers of PD that are not confounded by a specific cognitive demand.

## 2. Methods

EEG and pupillometry data from participants, including HC and those with PD, were obtained from a prior study [40]. Birkholz [40] used EEG and pupillometry to investigate differences in sustained ATTN and WM between young adults, older adults, and individuals with PD during a Continuous Performance Task (CPT), followed by a surprise recognition task to measure ATTN lapses and a visuospatial Change Detection Task (CDT) to assess WM capacity in different conditions. All participants provided written informed consent, in accordance with the guidelines of the North Dakota State University (NDSU) Institutional Review Board (Protocol IRB0004611). The final EEG and pupillometry recording for the attention portion of the study included *n* = 34 HC and *n* = 35 PD participants. HC participants were age-matched to the PD group to minimize age-related confounds in EEG and cognitive task performance.

**Figure 1:**
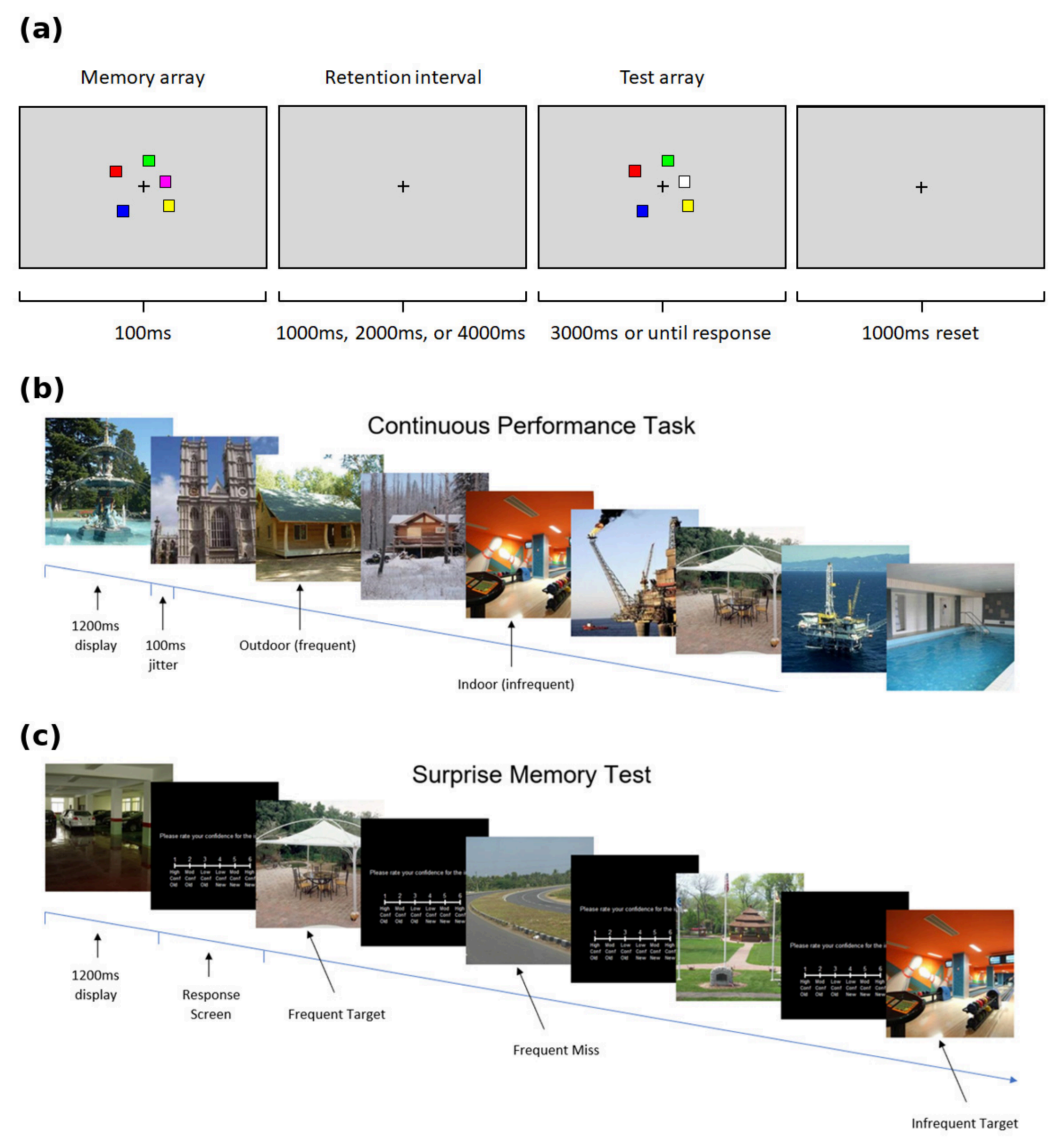
Panel (**a**) displays the CDT that assessed visual WM by presenting a memory display of 2–5 colored squares, followed by a variable delay (1000, 2000, or 4000 ms). Participants were then shown a test display and asked to indicate whether it matched the original. Panel (**b**) displays the CPT, which measured sustained attention by requiring participants to rapidly respond to a stream of scene images and press different keys for frequent and infrequent categories. Panel (**c**) depicts the surprise memory task embedded within the CPT, in which participants were unexpectedly asked to recall target stimuli from the attention trials, allowing assessment of incidental memory during sustained attention performance.

For the CPT, participants viewed 800 scene images for 1200 ms each (±100 ms jitter) with no interstimulus interval. Images were either from a frequent category (e.g., indoor; 700 images) or an infrequent category (e.g., outdoor; 100 images), counterbalanced across participants (Figure 1a). They responded using the left or right arrow keys, depending on the image type, aiming for both speed and accuracy. After a brief filler task, participants were given a surprise recognition memory test consisting of 300 images (200 old, 100 new), each followed by a confidence rating on a 6-point scale (1 = High Confidence Old to 6 = High Confidence New) (Figure 1b). These ratings were used to categorize memory trials based on both accuracy and confidence for downstream analysis. For the WM task, participants viewed 2–5 colored squares, followed by a variable delay (1000, 2000, or 4000 ms). This was followed by a test display in which participants were asked to determine whether it matched the original. See Figure 1 for both CPT (Figure 1a) and CDT (Figure 1b) layouts.

EEG was recorded using a 64-channel BioSemi ActiveTwo system (BioSemi, Amseterdam, The Netherlands) (sampling rate of 512 Hz), with electrodes positioned according to the modified 10–20 system. Data were referenced offline to the average of the left and right mastoids. Eye-tracking and pupil data were simultaneously recorded at 500 Hz using an EyeLink 1000 infrared eye tracker (SR Research Ltd. Ottawa, ON, Canada). A chin rest stabilized the head position throughout the task.

Preprocessing was conducted using MATLAB’s RB2024b EEGLAB and ERPLAB (MathWorks, Inc., Natick, MA, USA). Signals were bandpass filtered (0.1–40 Hz), downsampled to 256 Hz, and cleaned using ICA to remove ocular and muscular artifacts. Data were epoched from −200 to 1000 ms around each stimulus onset and baseline corrected. Only artifact-free epochs with accurate behavioral responses were included. Pupil data were preprocessed using custom MATLAB R2024B scripts. Blinks were linearly interpolated, and data were smoothed using a 5-sample moving average. Pupil diameter was baseline corrected to the 200 ms prestimulus interval. Trials with poor calibration or more than 25% missing data were excluded from the analysis. EEG and pupillary data were then separated into six bins, preserving the temporal structure of cognitive processing. For the CDT, data were binned by task phase (memory encoding vs. retrieval), set size (2, 3, 4, or 5 items), delay duration (1000, 2000, 4000 ms), and response accuracy (correct vs. incorrect), yielding 48 task-relevant bins that captured variations in cognitive load and memory maintenance demands. For the CPT, data were binned by participants’ memory performance (remembered vs. forgotten) and confidence ratings (low, moderate, high), enabling analysis of neurophysiological responses as a function of attentional accuracy and subjective certainty.

EEG and pupillometry data from each task were then processed using a standardized, custom-developed feature extraction toolbox in MATLAB [41]. The toolbox captures over 108 EEG and pupillometry features across various domains. Features were extracted for each predefined bin that corresponded to task-related periods, ensuring that the resulting measures were directly linked to cognitive processing stages. The results of the toolbox are bin x feature matrices that are then saved for each participant. Feature categories (e.g., Time-Domain, Frequency-Domain, ERP Components; Table 1) were assigned based on established conventions from the EEG and pupillometry literature and the toolbox’s design documentation [41], rather than on empirical loading patterns or task-specific extraction bins. Refer to Table 1 for a comprehensive list of all 108 features by category.

**Figure 2:**
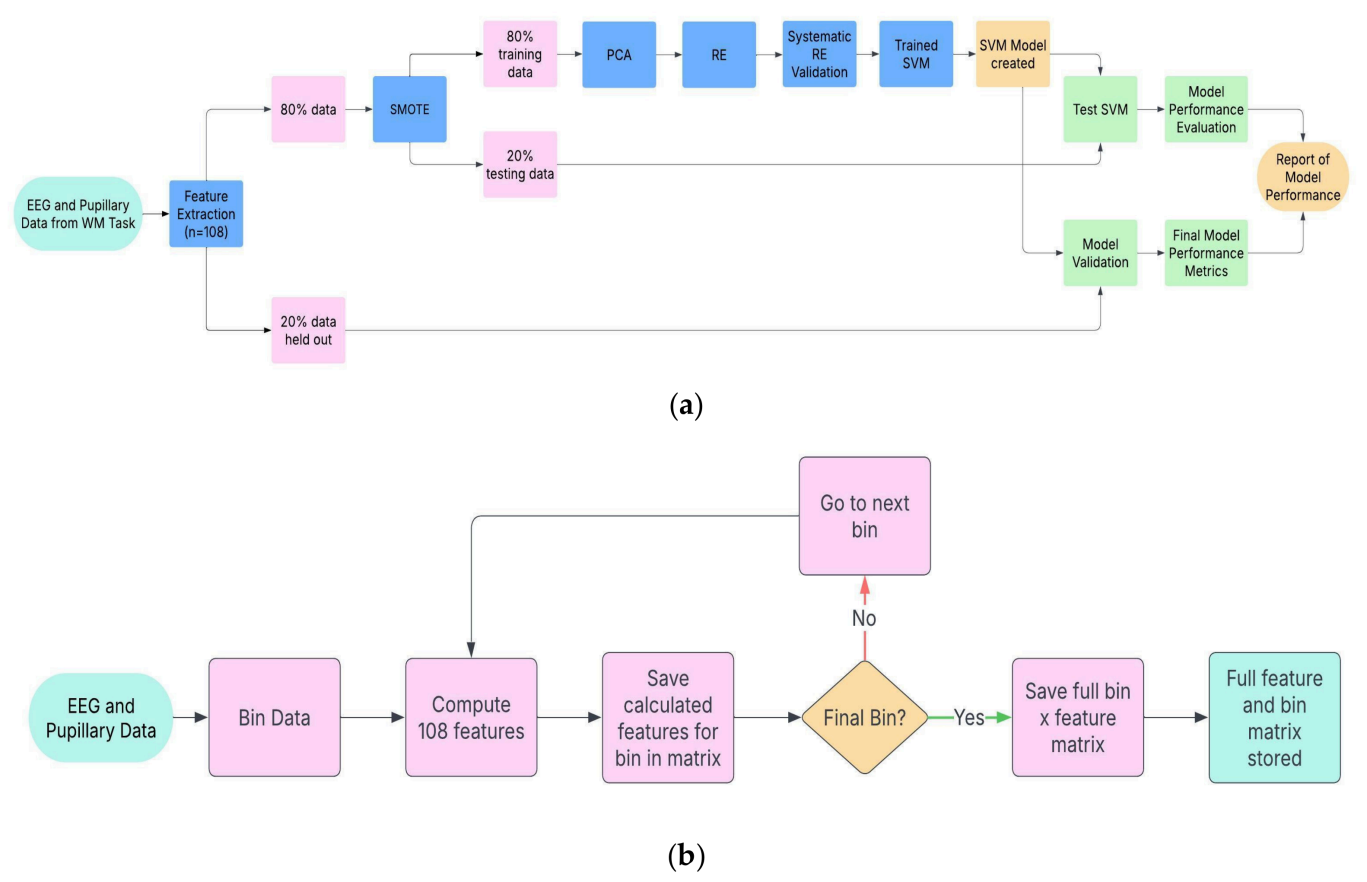
Overview of the machine learning (ML) pipeline and feature extraction process for classifying Parkinson’s disease (PD) from EEG and pupillometry data. (**a**) ML pipeline: EEG and pupillometry data are processed using a standardized, temporally aligned toolbox during a working memory change-detection task (WM-CDT) to capture 108 neural and physiological features per bin. Dimensionality reduction is performed using principal component analysis (PCA) with automated elbow point detection, followed by recursive elimination (RE) to identify the most informative components. The optimized feature set is then used to train a support vector machine with a radial basis function (SVM-RBF) kernel, validated on both test and hold-out datasets. (**b**) Detailed view of the feature extraction loop: data are segmented into bins; features are computed for each bin using custom MATLAB scripts and saved iteratively into a bin × feature matrix until all bins are processed. This approach enables time-resolved analysis of cognitive signatures in PD.

The bin x feature matrices from the feature extraction toolbox were then used in a downstream ML model, following a nested cross-validation design intended to prevent information leakage between model development and final evaluation. First, 20% of the data for each task was randomly set aside as a final hold-out set; these samples were not used in any subsequent step of model development, including class balancing, dimensionality reduction, feature selection, or model training, and were reserved exclusively for final model evaluation. The remaining 80% of the data (the development set) was used for model development via 5-fold cross-validation. Within each of the five outer folds, the development set was split into a training fold (80%) and an internal test fold (20%). The Synthetic Minority Oversampling Technique (SMOTE) was applied to balance classes using only the training fold in each split; the corresponding internal test fold was not exposed to SMOTE. Dimensionality reduction was then performed within the training fold using principal component analysis (PCA), followed by an automated elbow detection method to identify the optimal number of principal components. Recursive elimination (RE) was subsequently applied, also within the training fold, to further reduce dimensionality and retain only the most informative, non-redundant features. The resulting principal component and feature subset were used to train a support vector machine with a radial basis function kernel (SVM-RBF), which was then evaluated on the corresponding internal test fold. This process was repeated across all five outer folds, and the reported test-set accuracy, precision, recall, and F1-score reflect the average performance across these five folds. Once the optimal pipeline configuration (PCA components, RE-selected features, and model hyperparameters) was finalized using the development set, the final model was retrained on the full development set and evaluated once on the untouched hold-out set to provide an unbiased estimate of generalization performance. We also generated confusion matrices to examine differences in false-positive (FP) and false-negative (FN) classifications across models. We then examined which original features, and in which task bins they occurred, contributed most strongly to classification performance in both the WM and ATTN tasks. Refer to Figure 2 for a schematic of the complete feature extraction and nested cross-validation classification pipeline. To provide independent, model-agnostic validation of the PCA/RE-selected top features, we conducted univariate group-comparison analyses (independent-samples *t*-tests or Mann–Whitney U tests, as appropriate) comparing PD and HC groups on each of the 108 extracted features separately for the WM and ATTN tasks. Effect sizes are reported as Cohen’s d, with False Discovery Rate (FDR) correction applied to adjust for multiple comparisons.

## 3. Results

For the WM task, the automated elbow method identified 22 PCs and 10 original features as the optimal cutoffs, indicating that components beyond these contributed only marginal additional variance, as defined by the automated elbow algorithm’s identification of the inflection point where added components no longer produced meaningful gains in explained variance. RE determined that the highest model performance was achieved using 14 PCs and the top 7 PCA-weighted features. The resulting model yielded an accuracy of 71%, a precision of 0.679, a recall of 0.669, and an F1 score of 0.701 on the test set. Performance declined as fewer components were used, confirming the stability of this configuration. Final validation on the hold-out dataset resulted in 63% accuracy, a precision of 0.632, a recall of 0.619, and an F1 score of 0.626. The top features included frontal alpha asymmetry (FAA), prefrontal alpha index (PAI), alpha/theta ratio, ERP latency, alpha/beta ratio, half recovery time (pupillometry), and delta peak frequency, each extracted from bins with the largest PD–HC variance.

For the CPT-based ATTN task, the elbow point for PCs occurred at 21 components, and for PCA-loaded features at 12 features. RE optimization yielded the best performance with 14 PCs and 10 top features, producing 73% accuracy, a precision of 0.853, a recall of 0.591, and an F1 score of 0.699 on the test set. Final hold-out validation resulted in 70% accuracy, a precision of 0.760, a recall of 0.528, and an F1 score of 0.623. The top features included prefrontal alpha index (PAI), ERP entropy, theta/beta ratio, frontal alpha asymmetry (FAA), skewness, N200 features, gamma/beta ratio, and spike frequency.

**Figure 3:**
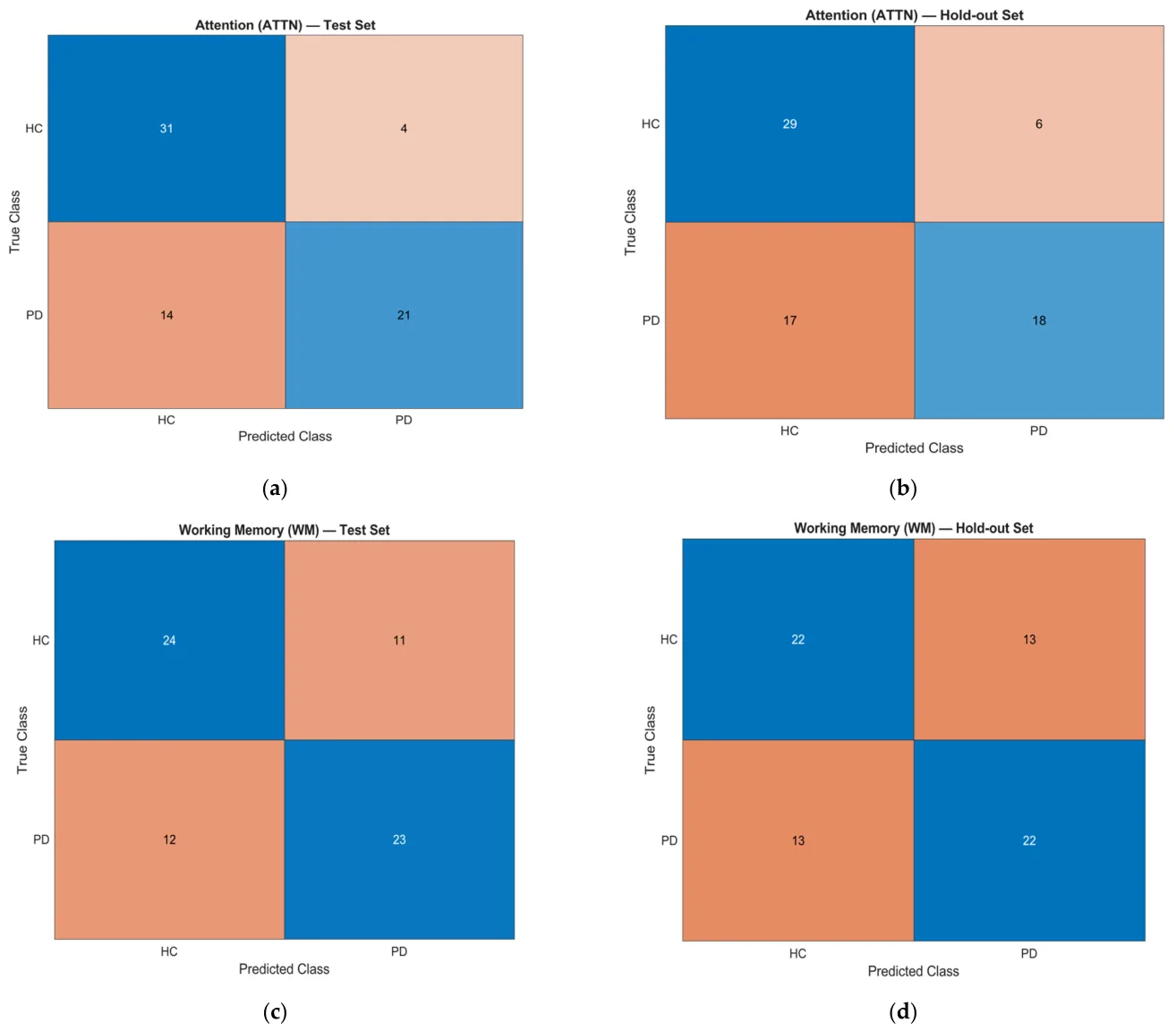
Confusion matrices for classification of Parkinson’s disease (PD) versus healthy controls (HC) using the working memory (WM) and attention (ATTN) models. Cell shading indicates classification outcome: blue cells denote correct classifications (true positives and true negatives), and orange cells denote misclassifications (false positives and false negatives). (**a**) WM test set. (**b**) WM hold-out set. (**c**) ATTN test set. (**d**) ATTN hold-out set. Each matrix is accompanied by the model’s classification performance metrics: WM—test accuracy = 71%, precision = 0.679, recall = 0.669, F1 = 0.701; hold-out accuracy = 63%, precision = 0.632, recall = 0.619, F1 = 0.626. ATTN—test accuracy = 73%, precision = 0.853, recall = 0.591, F1 = 0.699; hold-out accuracy = 70%, precision = 0.760, recall = 0.528, F1 = 0.623. The WM model demonstrated higher recall across both datasets, indicating greater sensitivity for detecting PD cases, whereas the ATTN model showed higher precision and overall accuracy, reflecting greater specificity. Together, these results highlight complementary strengths between the WM and ATTN paradigms, suggesting that a combined or hybrid approach may improve classification robustness for PD cognitive assessment.

The ATTN model achieved slightly higher test set accuracy (73% vs. 71%) and hold-out accuracy (70% vs. 63%) than the WM model, indicating better generalization to unseen data. The ATTN task also produced notably higher precision in both the test set (0.853 vs. 0.679) and hold-out set (0.760 vs. 0.632), suggesting fewer FP when identifying PD participants. However, the WM model demonstrated higher recall in both datasets (test set: 0.669 vs. 0.591; hold-out: 0.619 vs. 0.528), indicating greater sensitivity in detecting PD cases. F1-scores were comparable across tasks (WM: 0.701 test, 0.626 hold-out; ATTN: 0.699 test, 0.623 hold-out), reflecting a balanced trade-off between precision and recall in both paradigms. Consistent with these trends, the ATTN model showed a higher number of FN than the WM model across both datasets, whereas the WM model produced more FP. This pattern indicates that ATTN favored specificity, while WM favored sensitivity, reflecting complementary error profiles across the two paradigms. See Table 2 for a comparison of model performance metrics and top features between tasks and Figure 3 for the confusion matrices for both tasks.

Univariate analyses provided independent support for several top-ranked features. For the ATTN task, PAI (d = −0.65, *p* = 8.5 × 10^−4^, FDR-adjusted *p* = 0.027), theta/beta ratio (d = 0.63, *p* = 0.0010, FDR-adjusted *p* = 0.027), skewness (d = −0.61, *p* = 0.0020, FDR-adjusted *p* = 0.036), N200 amplitude (d = −0.51, *p* = 0.0071), spike frequency (d = 0.49, *p* = 0.0093), ERP entropy (d = −0.48, *p* = 0.0115), N200 average amplitude (d = −0.44, *p* = 0.022), and FAA (d = 0.43, *p* = 0.025) all showed moderate group differences. Gamma/beta ratio, by contrast, showed a near-zero univariate effect (d = −0.088) despite contributing substantial weight to the leading principal components, indicating that PCA-based ranking captures shared covariance structure across features rather than simply reflecting standalone group differences; this feature is better interpreted as multivariate-relevant than as an independent biomarker. For the WM task, oscillatory-ratio and spectral-parameterization features showed moderate-to-large effects, including alpha/theta ratio (d = 0.779, *p* = 0.0028), alpha/beta ratio (d = 0.405, *p* = 0.106), and FOOOF-derived alpha peak height at frontal and posterior sites (d = 0.84–1.01, *p* = 0.0009–0.0088), while signal latency (d = 0.213) and pupillary half recovery time (d = 0.117) showed smaller effects. Notably, PAI (d = −0.455, *p* = 0.110) and FAA (d = 0.058, *p* = 0.817) did not reach univariate significance in the WM task, despite ranking among its top PCA/RE-selected features and showing significant univariate effects in the ATTN task. As with gamma/beta ratio in ATTN, this dissociation suggests these features’ contribution to WM classification is driven by their role in the shared multivariate covariance structure rather than independent group-level separability in this task, indicating that multivariate relevance and univariate discriminability can differ not only across features, but across tasks for the same feature. Together, these results indicate that most top-ranked features show independent, if variable, group-level separability, though not all survived correction for multiple comparisons, underscoring that multivariate ranking and univariate discriminability are related but distinct properties.

Among the top-selected features, PAI and FAA were among the most influential in both tasks by PCA/RE ranking. Univariate analyses supported this pattern in the ATTN task, where both features showed statistically significant group differences, but not in the WM task, where neither reached univariate significance despite their multivariate contribution. This task-dependent dissociation suggests PAI and FAA’s value as cross-task biomarkers may be more robust in attention-related than working-memory-related contexts, warranting further investigation with formal feature-importance methods [42,43]. WM-specific top features reflected frontal network engagement and low-frequency oscillations (e.g., delta peak frequency, alpha/theta ratio, half recovery time) [11,44]. In contrast, ATTN-specific features emphasized variability and synchrony measures (ERP entropy, skewness, theta/beta ratio, spike frequency, and gamma/beta ratio), indicating a greater weighting on dynamic attentional processes [6,23,35].

## 4. Discussion

This study compared the performance of ML classification models using an SVM-RBF trained on EEG and pupillary features from a WM and ATTN paradigm to classify PD from HC. By using an identical feature-extraction and classification pipeline, we evaluated performance differences and identified both shared and task-specific biomarkers of cognitive dysfunction in PD.

### 4.1. Model Performance Comparison

**Figure 4:**
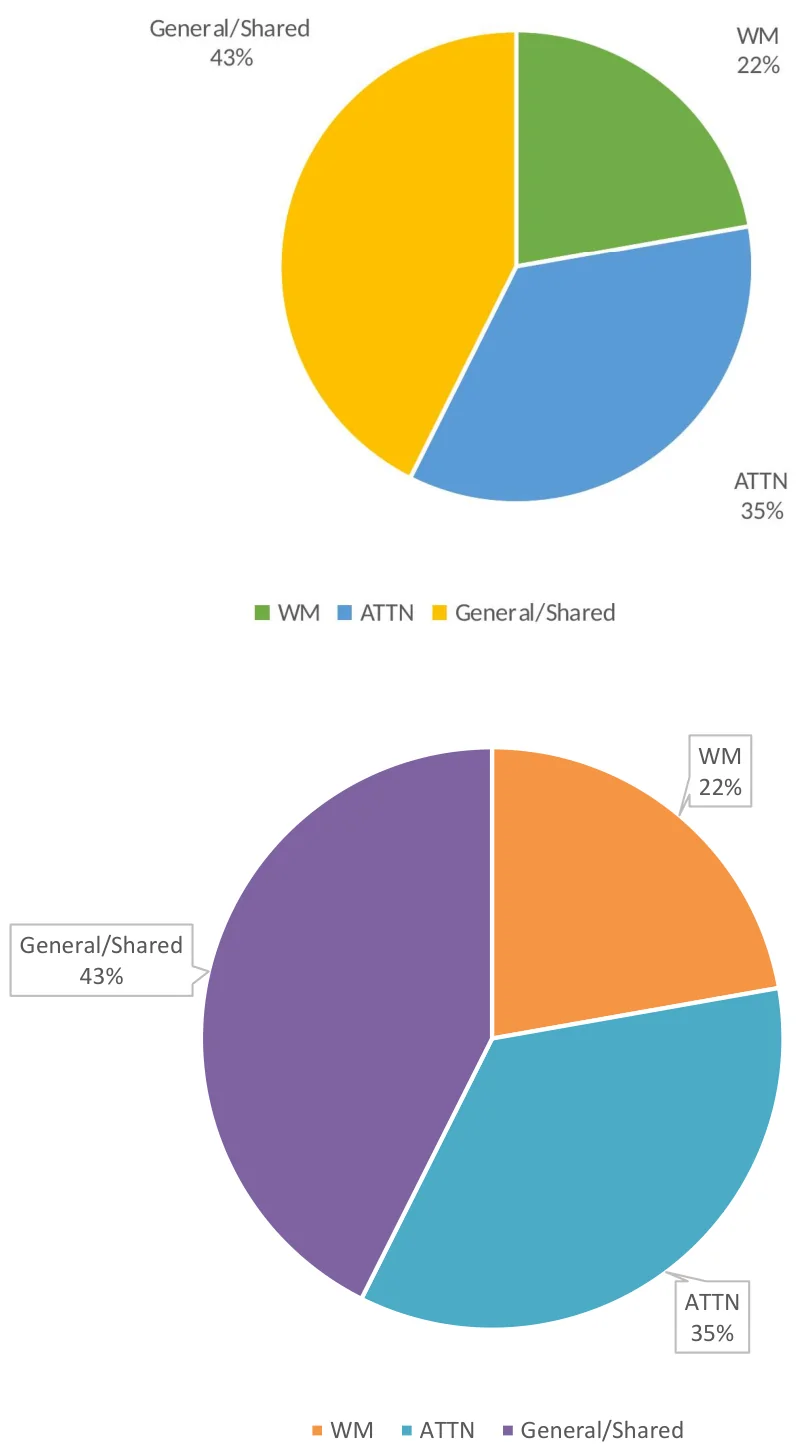
Distribution of the 108 extracted EEG and pupillometry features categorized by their likely cognitive association. Features were classified as primarily associated with sustained attention (38 features), working memory (24 features), or general/shared relevance across tasks (46 features), based on established neurophysiological and behavioral literature. Attention-related features included ratios such as theta/beta and gamma/beta, synchrony measures, and pupillometry dynamics linked to vigilance and arousal regulation. Working memory-related features included alpha/theta ratio, delta peak frequency, and ERP components associated with encoding and retrieval. The disproportionate representation of attention-related features may have contributed to the higher precision and accuracy observed in the ATTN model compared to the WM model.

The WM and ATTN classification models confirmed differences in performance profiles despite being trained using the same feature extraction and ML pipeline. The ATTN model achieved higher overall accuracy than the WM model in both the test set (73% vs. 71%) and the hold-out set (70% vs. 63%), suggesting that the ATTN paradigm may generalize more effectively to unseen data. The ATTN model also exhibited high precision, indicating greater specificity in PD classification and fewer FPs. In contrast, the WM model achieved higher recall across both tests and the hold-out set, suggesting greater sensitivity to PD-related cognitive differences. High recall of the WM model means that more PD cases were successfully detected but also resulted in more FPs. Conversely, the ATTN model’s higher precision means that when it predicted PD, it was correct more often, but it also missed more true PD cases. This contrast in performance could result from the selected set of task-dependent feature profiles extracted, which were present in the extraction toolbox. Across the 108 extracted features, these features could be geared more towards ATTN than WM, enabling the model to generalize better. When examining the selected features, a high percentage of features are shared between WM and ATTN (*n* = 46; 42.6%), whereas a higher percentage of features (*n* = 38; 35.2%) were primarily ATTN measures, rather than WM measures (*n* = 24; 22.2%). See Figure 4 for a breakdown of feature measure percentages across tasks and those shared between tasks. The higher proportion of ATTN-related features in the extraction toolbox likely enhanced both feature discriminability and classification-relevant signal strength for the ATTN paradigm. Many of these features are strongly modulated by sustained attention and vigilance demands, leading to more distinct and separable patterns between PD and HC during ATTN trials. This increased discriminability reduced the false positive rate (FP = 11.4% in test; 17.1% in hold-out) compared to WM (FP = 31.4% in test; 37.1% in hold-out), allowing the model to identify true PD cases with greater specificity and thereby elevating precision.

In contrast, the WM paradigm had fewer task-specific features, which may have diluted classification-relevant signal strength and contributed to a higher FP rate, despite its advantage in recall. WM’s higher recall corresponds to a lower false-negative rate (FN = 34.3% in test; 37.1% in hold-out) than ATTN (FN = 40.0% in test; 48.6% in hold-out), suggesting that WM features are sensitive to subtle PD-related changes in memory encoding and maintenance processes that persist even when behavioral accuracy is preserved. From a clinical perspective, FN are problematic because they represent missed PD cases, delaying diagnosis and treatment. Minimizing FN is critical in screening contexts where the goal is to detect as many true cases as possible. FP, on the other hand, indicate healthy individuals incorrectly flagged as having PD, which can lead to unnecessary anxiety, testing, and healthcare costs. Reducing FP is more important in confirmatory diagnostic settings where precision is prioritized over broad sensitivity.

In this study, the WM model’s lower FN rate raises the hypothesis that WM-based classification may be better suited for screening and early-detection applications, where minimizing missed PD cases is prioritized. Conversely, the ATTN model’s lower FP rate raises the hypothesis that ATTN-based classification may be better suited for confirmatory-testing applications, where minimizing false alarms is prioritized. Given the modest sample size (*n* = 69) and the absence of an independent external validation cohort in this study, we frame this screening-versus-confirmatory distinction as a hypothesis for future validation in larger, externally validated samples, rather than as a direct clinical implication at this stage.

It is important to explicitly consider whether classification performance in the 63–70% range is practically useful, and in what contexts. At present, this performance level is not sufficient to support standalone diagnostic use; PD diagnosis relies on established clinical criteria, and a classifier with a 30–37% error rate would not meet the accuracy needed for confirmatory diagnosis in isolation. In its current form, this pipeline is best understood as a research tool for biomarker discovery, useful for identifying candidate task-linked EEG and pupillometry features and comparing their discriminative value across cognitive paradigms, rather than as a tool ready for clinical deployment. With further development, including training on larger and more diverse datasets, external validation across independent cohorts, and refinement of the feature set and modeling pipeline, this approach could plausibly be developed into practical tools for adjunctive screening (flagging individuals for closer clinical follow-up) or longitudinal monitoring (tracking within-person changes in classification-relevant features over time). At this stage, however, these represent potential future applications contingent on substantial additional validation, not capabilities of the current model.

### 4.2. Feature Level Comparison

Two features, PAI and FAA, were shared across both tasks, suggesting differences in frontal activity in individuals with PD for both WM and ATTN. Their presence in both paradigms implies that certain oscillatory balance and asymmetry patterns may reflect a stable neural phenotype of PD, detectable regardless of specific cognitive demands. This interpretation is supported by prior work showing that alterations in frontal alpha asymmetry and related oscillatory dynamics are linked to PD-related deficits in attention, executive function, and motor control, and can be detected even in resting-state EEG recordings, independent of specific task paradigms [42,44]. In addition to electrophysiological evidence, neuroimaging and neuropathological studies have demonstrated dopaminergic asymmetry in frontostriatal circuits in PD, with reduced dopamine transporter binding and receptor availability preferentially affecting one hemisphere. Such asymmetry has been associated with lateralized motor symptoms, altered cortical activation patterns, and cognitive differences, particularly in executive and attentional control [43].

From a modeling perspective, these shared features may act as baseline discriminators, providing a consistent classification anchor across tasks and reducing reliance on task-specific variability. However, their relative weights on the top PCs were slightly different across tasks, being somewhat higher in ATTN (PAI 3.24; FAA 3.04) than in WM (PAI 2.5; FAA 2.75). This difference in weights could account for variations in model performance. From a clinical perspective, the presence of both FAA and PAI across paradigms, despite their univariate significance being confirmed only in ATTN, supports their potential as candidate EEG biomarkers worth further investigation, though their differing task-dependent univariate profiles indicate this candidacy is currently stronger for ATTN than for WM. Additionally, since these features appeared across task types, they are not strictly bound to specific task phases. They could be leveraged in a shorter, more flexible testing protocol, which is advantageous for the PD population, which experiences fatigue-related limitations.

### 4.3. Implications for Using Task-Based Biomarkers for PD Diagnosis and Treatment

These findings have several important implications for PD research, diagnosis, and treatment. The development and application of a high-dimensional, temporally aligned EEG-pupillometry feature extraction toolbox demonstrate the feasibility of capturing a broad range of neurophysiological signals often missed by traditional pipelines that focus on small feature spaces. This richer signal space can help improve classification performance, support biomarker discovery by revealing discriminative patterns tied to specific cognitive events and enhance interpretability by allowing researchers to map features back to their underlying neural and physiological processes. The identification of cross-task biomarkers suggests the presence of stable neural signatures in PD that transcend specific task demands and cognitive domains. These consistently expressed features could form the basis of a streamlined cognitive monitoring task designed specifically to elicit the neural and physiological responses most sensitive to PD. Such a task could be shorter, easier to administer, and less cognitively taxing than full WM or ATTN paradigms, making it more feasible for routine clinical use and remote monitoring. By focusing on the identification of robust, high-yield biomarkers, such designs could increase patient compliance, reduce the testing burden, and facilitate longitudinal tracking of disease progression and treatment efficacy.

The complementary strengths of the WM and ATTN models highlight trade-offs in clinical application. From a diagnostic standpoint, higher recall in WM may be advantageous for screening, minimizing FN, and ensuring that at-risk individuals are identified early. In contrast, higher precision in ATTN may be more valuable for confirming diagnoses and reducing FPs, which is important for therapeutic decision-making and trial enrollment. As noted above, these potential roles are hypothesis-generating given the current sample size and validation scope, and would need to be confirmed in larger, externally validated cohorts before informing actual screening or diagnostic protocols. From a treatment standpoint, the ability of the proposed EEG and pupillometry framework to extract stable, task-linked features is directly relevant to evaluating the outcomes of cognitive intervention therapy in PD. By providing objective, temporally precise biomarkers that are sensitive to subtle changes in ATTN and WM, this approach could quantify improvements or declines in targeted cognitive domains following therapy. Because these features are consistent across different cognitive paradigms, they offer a way to detect generalized transfer effects beyond the trained tasks, while also identifying domain-specific gains. This could help researchers and clinicians determine not only whether an intervention is effective, but also how it modifies underlying neural processes, enabling more personalized and adaptive treatment strategies over time.

Collectively, the results underscore the potential of integrating multi-task, multimodal biomarker assessments into both research and clinical workflows. In PD research, such approaches could advance understanding of cognitive phenotypes and their neural underpinnings. Clinically, they could support earlier detection, track longitudinal changes, and provide objective endpoints for evaluating therapeutic efficacy. The ability to detect subtle cognitive changes with interpretable models means these biomarkers could serve as sensitive indicators of treatment response in clinical trials and ongoing care, enabling real-time monitoring of therapeutic impact and allowing clinicians to adjust interventions proactively. Ultimately, integrating these objective, task-linked biomarkers into clinical workflows has the potential to enhance PD care by shifting cognitive assessment from infrequent, subjective snapshots to continuous, interpretable monitoring that not only improves diagnostic precision but also drives more responsive, personalized, and effective therapeutic strategies.

## 5. Conclusions

This study compared ML models trained on EEG and pupillometry features from WM and ATTN paradigms to classify PD versus HC. Using an identical feature-extraction and PCA-based dimensionality-reduction pipeline, we identified both shared and task-specific features that contributed to model performance. The ATTN model achieved higher accuracy and precision, indicating greater specificity, while the WM model achieved higher recall, reflecting greater sensitivity in detecting PD-related cognitive differences.

These complementary strengths suggest that combining WM and ATTN paradigms could yield more robust and generalizable cognitive biomarkers for PD. Moreover, the identification of cross-task features such as PAI and FAA highlights their potential as core neurophysiological markers. By refining these models and integrating them into clinical workflows, task-based neurophysiological assessment could become a scalable and objective tool for early detection and monitoring of cognitive impairment in PD.

## 6. Limitations and Future Work

While this study provides a promising foundation for cognitive dysfunction biomarker discovery and classification, several limitations warrant attention. First, the sample size (*n* = 69) limits generalizability and may inflate model performance estimates; relatedly, the ratio of 108 extracted features to 69 participants is small relative to the feature space, increasing overfitting risk despite PCA, recursive elimination, and nested cross-validation. These steps reduce, but do not eliminate, this risk, and reported metrics should be interpreted accordingly. Larger, multi-site samples are needed for more stable feature selection and reliable generalization estimates.

Second, the 108-feature set, while relatively large and diverse, may not capture all relevant neurophysiological or behavioral markers of PD-related cognitive change, and skewed slightly toward ATTN- over WM-relevant measures, which may partly explain the performance differences between tasks. Spectral feature interpretation is also limited by the frequent absence of clear peaks outside the alpha band, complicating ratio- and peak-based metrics; spectral parameterization methods that separate oscillatory from aperiodic (1/f) activity offer a promising solution for future work.

Third, this study examined WM and ATTN only; future work should incorporate executive-function tasks for a more comprehensive assessment of cognitive impairment in PD.

Fourth, while the bin × feature structure supports interpretability by linking features to task stages, future work should use SHAP to formally verify feature importance, and could pursue AI-driven models designed specifically for clinical interpretability to support trust and integration into healthcare workflows.

Fifth, the current pipeline is not designed for real-time or point-of-care use. Preprocessing (ICA-based artifact removal, PCA/RE, cross-validated model selection) is computationally intensive and applied offline at the group level; reported performance reflects post hoc analysis, not live single-recording inference. Clinical translation would require automated artifact handling, a fixed pre-validated model, and prospective testing on streaming single-patient data. The present findings should be read as establishing feasibility for offline biomarker discovery, not readiness for real-time clinical deployment.

Sixth, medication status (e.g., on- vs. off-medication testing) and disease duration/severity (e.g., UPDRS scores) were not recorded or controlled for in the PD group. Both are established confounders in PD EEG and cognitive research—dopaminergic medication modulates cortical oscillatory activity and cognitive performance, and disease duration/severity tracks with progressive neurophysiological and cognitive change—so we cannot rule out their contribution to the observed classification performance independent of the WM- and ATTN-related features of interest. Future studies should record, and where possible control for, these variables to better isolate cognitively specific biomarkers from clinical confounds.

Taken together, these limitations point to a clear roadmap for future work. The most immediate priority is expanding to larger, multi-site samples with independent external validation cohorts, which would address the sample-size-to-feature ratio, allow more stable feature selection, and provide a genuine test of generalizability beyond the current hold-out set. In parallel, incorporating medication status and disease duration/severity as recorded covariates would allow these established clinical confounders to be statistically accounted for rather than left unmeasured. Once a larger validated dataset is available, formal, model-agnostic feature-importance methods (e.g., SHAP, permutation importance) should be applied to move beyond PCA-weight and univariate-effect-size evidence toward independently validated biomarker claims. Extending the pipeline to additional cognitive domains, particularly executive function, would test whether the cross-task consistency observed for WM and ATTN generalizes further, while integrating spectral parameterization methods would refine the interpretability of oscillatory features. Only after these steps are complete would translating the pipeline toward real-time, point-of-care deployment become a reasonable next stage, since the current pipeline is a research tool for offline biomarker discovery rather than a system designed for live clinical inference. Pursued in this order, from validation and confound control, to formal importance testing, to task generalization, to eventual clinical translation, this line of work could move task-based EEG and pupillometry biomarkers from proof-of-concept toward tools with genuine adjunctive screening or monitoring utility in PD.

## Figures and Tables

**Table 1:** All 108 EEG and pupillometry features used in the toolbox.

Category	(n)	Features
Time-Domain	21	Mean amplitude, Standard deviation of amplitude, Peak-to-peak amplitude, Root mean square amplitude, Variance of amplitude, Zero crossings, Skewness, Kurtosis, Maximum peak amplitude, Minimum peak amplitude, Minimum signal value, Maximum signal value, Signal latency, Area under the curve, Line length of waveform, Signal energy, Signal power, Noise power, Signal-to-noise ratio, Autocorrelation at lag 1, Interquartile range of amplitude
Hjorth Parameters	4	Hjorth activity, Hjorth mobility, Hjorth complexity, Hjorth complexity/mobility ratio
Frequency-Domain	19	Delta band power, Theta band power, Alpha band power, Beta band power, Gamma band power, Relative band power, Delta peak frequency, Theta peak frequency, Alpha peak frequency, Beta peak frequency, Gamma peak frequency, Alpha/Beta power ratio, Theta/Beta power ratio, Alpha/Theta power ratio, Gamma/Beta power ratio, Mean frequency, Median frequency, Petrosian fractal dimension, Burst percentage
Pupillometry	15	Mean pupil diameter, Standard deviation of pupil diameter, Minimum pupil diameter, Maximum pupil diameter, Range of pupil diameter, Variance of pupil diameter, Mean pupil velocity, Mean pupil acceleration, Blink rate, Blink durations, Mean blink duration, Task-evoked pupillary response latency, Task-evoked pupillary response peak amplitude, Half recovery time of pupil response, Area under the curve of task-evoked pupillary response
ERP Components	12	N100 amplitude, N100 latency, N200 amplitude, N200 latency, P200 amplitude, P200 latency, P300 amplitude, P300 latency, Average N200 amplitude, Average P300 amplitude, Component amplitude ratio, P300 variability
Phase and Synchrony Metrics	10	Theta synchrony, Alpha desynchronization, Phase difference, Phase synchrony, Phase resetting index, Alpha band coherence, Beta band coherence, Phase locking value, Phase locking value mean, Cross-channel coupling
Entropy and Complexity	7	ERP entropy, Tsallis entropy, Hurst exponent, Teager energy operator signal, Mean Teager energy operator, Global field power, Root mean square of successive differences
Spike and Waveform Features	5	Waveform sharpness, Spike frequency, Spike amplitude, Spike/slow wave amplitude ratio, Slow wave activity
Asymmetry and Indices	3	Frontal alpha asymmetry, Prefrontal asymmetry index, Phase-amplitude coupling
Spectral Features	12	Spectral centroid, Spectral entropy, Spectral flatness, Spectral roll-off frequency, Spectral kurtosis, Spectral variability, Spectral edge frequency (95%), Event-related spectral perturbation, Power spectral density (PSD) slope, PSD skewness, PSD kurtosis, Cumulative spectral power

**Table 2:** Comparison of Working Memory and Sustained Attention Classification Model Performance.

Task	Set	Accuracy (%)	Precision	Recall	F1	Top Features
Working Memory (WM)	Test Set	71	0.679	0.669	0.701	FAA, PAI, alpha/theta ratio, ERP latency, alpha/beta ratio, half recovery time (pupillometry), and delta peak frequency
	Hold-out Set	63	0.632	0.619	0.626	
Sustained Attention (ATTN)	Test Set	73	0.853	0.591	0.699	PAI, ERP entropy, theta/beta ratio, frontal FAA, skewness, N200 features, gamma/beta ratio, and spike frequency
	Hold-out Set	70	0.760	0.528	0.623	

## Data Availability

The de-identified EEG and pupillometry datasets generated and analyzed during the current study are available from the corresponding author upon reasonable request. Access to de-identified raw data from human participants can be available to Jeffrey S. Johnson or Samuel A. Birkholz upon reasonable request. The custom feature extraction toolbox and scripts used for model training and analysis are openly available on GitHub at [https://github.com/mercedesterry/EEG-and-Pupillary-Feature-Extraction-Toolbox] (accessed on 20 July 2025).

## Abbreviations

The following abbreviations are used in this manuscript:

ATTN	Sustained Attention
CDT	Change Detection Task
CNN	Convolutional Neural Network
CPT	Continuous Performance Task
DT	Decision Tree
EEG	Electroencephalography
ERP	Event-Related Potential
FAA	Frontal Alpha Asymmetry
FN	False Negative
FP	False Positive
HC	Healthy Controls
ICA	Independent Component Analysis
MATLAB	Matrix Laboratory
ML	Machine Learning
NDSU	North Dakota State University
PAI	Prefrontal Alpha Index
PC	Principal Component
PCA	Principal Component Analysis
PD	Parkinson’s Disease
PSD	Power Spectral Density
RBF	Radial Basis Function
RE	Recursive Elimination
SHAP	Shapley Additive Explanations
SMOTE	Synthetic Minority Oversampling Technique
SVM	Support Vector Machine
SVM-RBF	Support Vector Machine with Radial Basis Function Kernel
WM	Working Memory
